# Lymphocyte–monocyte–neutrophil index: a predictor of severity of coronavirus disease 2019 patients produced by sparse principal component analysis

**DOI:** 10.1186/s12985-021-01561-9

**Published:** 2021-06-04

**Authors:** Yingjie Qi, Jian-an Jia, Huiming Li, Nagen Wan, Shuqin Zhang, Xiaoling Ma

**Affiliations:** 1 The First Affiliated Hospital of University of Science and Technology of China (Anhui Provincial Hospital Infection Hospital), Susong Road 218#, Hefei, 230022 Anhui Province China; 2 Department of Laboratory Medicine, The 901Th Hospital of Joint Logistics Support Force of Chinese People’s Liberation Army, Hefei, 230031 Anhui China; 3 Department of Laboratory Medicine, The First Affiliated Hospital of Nanchang University, Nanchang, 330006 Jiangxi China; 4 Centre for Computational Systems Biology, School of Mathematical Sciences, Fudan University, Shanghai, 200433 China

**Keywords:** SARS-CoV-2, COVID-19, Severity, Prediction, Principal component analysis

## Abstract

**Background:**

It is important to recognize the coronavirus disease 2019 (COVID-19) patients in severe conditions from moderate ones, thus more effective predictors should be developed.

**Methods:**

Clinical indicators of COVID-19 patients from two independent cohorts (Training data: Hefei Cohort, 82 patients; Validation data: Nanchang Cohort, 169 patients) were retrospected. Sparse principal component analysis (SPCA) using Hefei Cohort was performed and prediction models were deduced. Prediction results were evaluated by receiver operator characteristic curve and decision curve analysis (DCA) in above two cohorts.

**Results:**

SPCA using Hefei Cohort revealed that the first 13 principal components (PCs) account for 80.8% of the total variance of original data. The PC1 and PC12 were significantly associated with disease severity with odds ratio of 4.049 and 3.318, respectively. They were used to construct prediction model, named Model-A. In disease severity prediction, Model-A gave the best prediction efficiency with area under curve (AUC) of 0.867 and 0.835 in Hefei and Nanchang Cohort, respectively. Model-A’s simplified version, named as LMN index, gave comparable prediction efficiency as classical clinical markers with AUC of 0.837 and 0.800 in training and validation cohort, respectively. According to DCA, Model-A gave slightly better performance than others and LMN index showed similar performance as albumin or neutrophil-to-lymphocyte ratio.

**Conclusions:**

Prediction models produced by SPCA showed robust disease severity prediction efficiency for COVID-19 patients and have the potential for clinical application.

**Supplementary Information:**

The online version contains supplementary material available at 10.1186/s12985-021-01561-9.

## Background

Since December 2019, the novel Coronavirus Disease 2019 (COVID-19) outbreak, which occurred in Wuhan, Hubei province, China, has infected over 5.7 million people globally by May 29th, 2020 [1]. As this severe acute respiratory syndrome coronavirus 2 (SARS-CoV-2) spreads globally, great strains are put on health care system of every country. In order to save more lives, more concerns should be focused on severe ill patients, thus it is critical to recognize severe ill patients from mild ones. Possible risk factors for progressing to severe illness may include, but are not limited to, older age, and pre-existing chronic medical conditions such as lung disease, heart failure, cerebrovascular disease, and so on [2]. Clinically, the main symptoms of severe COVID-19 patients include fever, leukopenia, lymphopenia, thrombocytopenia, C-reactive protein increase, and cytokines abnormity [3–6]. Lactate dehydrogenase, interleukin 6, and D-dimer were also reported as risk factors for progression to severe status [7]. As such, plenty of clinical laboratory markers could be used to predict the severity of COVID-19 patients and it is challenging to utilize such rich laboratory indicators for clinical diagnosis and treatment.

Therefore, clinical characteristics and dozens of laboratory markers of 82 COVID-19 patients from the First Affiliated Hospital of University of Science and Technology of China were analyzed retrospectively and Sparse Principal Component Analysis (SPCA) was performed to examine the correlation between these markers and extract relevant features. Then the prediction models for disease severity were constructed based on logistic regression using the principal components (PCs) produced by SPCA. Prediction efficiency of these models was assessed and compared with classical blood markers. Furthermore, an independent cohort including 169 COVID-19 patients from the First Affiliated Hospital of Nanchang University was used as a validation dataset and prediction efficiency of these models was also evaluated.

## Methods

### Patients enrollment

In this study, 82 patients (Hefei Cohort) with confirmed COVID‐19 admitted to the First Affiliated Hospital of University of Science and Technology of China from January 23, 2020 to March 3, 2020 were enrolled. Independent cohort enrolled 169 COVID-19 patients (Nanchang Cohort) from the First Affiliated Hospital of Nanchang University from January 23, 2020 to March 10, 2020. This study was approved by the Ethics Committee of the First Affiliated Hospital of University of Science and Technology of China and the Ethics Committee of the First Affiliated Hospital of Nanchang University. According to Diagnosis and Treatment Protocol for Novel Coronavirus Pneumonia (Trial version 7) [8], released by National Health Commission & State Administration of Traditional Chinese Medicine, all of the patients were confirmed using fluorescent reverse transcription PCR, and divided into severe group and mild group. Adult patients meet any of the following criteria were classified into severe type: (1) Respiratory distress (≥ 30 breaths/min); (2) Oxygen saturation ≤ 93% at rest; (3) Arterial partial pressure of oxygen (PaO2)/fraction of inspired oxygen (FiO2) ≤ 300 mmHg ( l mmHg = 0.133 kPa). Cases with chest imaging that showed obvious lesion progression within 24–48 h > 50% should be managed as severe cases.

### Data collection

After all of the patients were discharged from hospital except one who died one day after admission to hospital, the clinical data of these patients were retrospected including demographic data, medical history, complete blood counts, blood biochemistry, coagulation indices, infection-related indices, and myocardial markers. Blood routine test, clinical chemistry markers, coagulation functions and T lymphocytes typing were tested on Mindray 6900 hematology analyzer, Beckmen 5800 automated biochemistry analyzer, Succeed SF8000 hemagglutinin analyzer and BD FACScalibur flow cytometer respectively. Infection-related indices and myocardial markers were detected on Roche cobas e601 automated electrochemical luminescence immunodetector. Since all of the patients have taken several laboratory tests, results of three time points during hospitalization were collected: the first time point upon hospitalization, the medium-term after hospitalization, and the last time of laboratory test before hospital discharge.

### Statistical analysis

Statistical analysis was performed using the R software, version 3.6.3. The results of continuous variables were expressed as the median with interquartile range and analyzed using Wilcoxon signed-rank test or Pearson correlation test. Categorical variables were presented as numbers (percentages) and analyzed using chi-squared test or Fisher's exact test. Repeated measured data of different time points was compared by repeated measures analysis of variance. Multivariate logistic regression analysis was adopted to identify risk factors of disease progression.

### Sparse principal component analysis (SPCA) and model evaluation

SPCA was performed using the R software package (sparsepca, https://github.com/erichson/spca) [9]. Clinical continuous variables of Hefei Cohort including age and all of the above laboratory indicators were used and the data were centered and scaled by subtracting each mean and dividing each standard deviation to allow all the variables to have unit variance. In the SPCA process, controlling parameter alpha was adjusted from 0.0001 to 0.002 with stepsize 0.0001 for better variable selection, and for each alpha value, the cumulative variance and number of variables selected in the top principal components (PCs) were calculated. PCs produced by SPCA were then subjected to multivariate logistic regression for disease severity prediction. The prediction models using PCs were evaluated using receiver operator characteristic curve (ROC) and the area under curve (AUC) was calculated. The accuracy, sensitivity, specificity, positive predictive value (PPV) and negative predictive value (NPV) were also calculated. For clinical net benefit assessment [10], decision curve analysis was also performed using rmda package ( http://mdbrown.github.io/rmda/).

#### Independent cohort validation

The 169 COVID-19 patients of Nanchang Cohort were used as an independent validation cohort. Using scaled clinical markers of each patient, the PCs of each patient were calculated according to the corresponding PC loadings matrix originated from Hefei Cohort. The produced prediction models were then used to predict the disease severity of this independent cohort and the prediction efficiency was estimated using ROC. The sensitivity, specificity, PPV, NPV and accuracy of each marker were also calculated. The clinical net benefit was evaluated using decision curve analysis.

## Results

### Demographics and baseline laboratory test results

The demographic and the first-time clinical laboratory test results of 82 COVID-19 patients in Hefei Cohort, are showed in Table 1. Compared with the 54 mild ill COVID-19 patients, most of the 28 severe ill patients are male and have comorbidities. Severe ill COVID-19 patients also showed older age, increased white blood cell count (WBC), neutrophil count (NEU), neutrophil percentage (NEU%), aspartate aminotransferase (AST), alanine aminotransferase (ALT), gamma-glutamyltransferase (GGT), glucose (Glu), Urea, lactic dehydrogenase (LDH), serum amyloid a (SAA), C-reactive protein (CRP), procalcitonin (PCT), interleukin-6 (IL-6), D-Dimer (DD), and myohemoglobin (MYO). Meanwhile the lymphocyte count (LYM) and lymphocyte percentage (LYM%), albumin (Alb), calcium (Ca), phosphorus (P), fibrinogen (FIB) levels of severe ill patients decreased significantly.

**Table 1:** Demographics and baseline laboratory markers of mild and severe ill COVID-19 patients in Hefei Cohort

	Mild (N = 54)	Severe (N = 28)	*P*
Gender = male (%)	28 (51.9)	23 (82.1)	0.015
Smoke = yes (%)	2 (3.7)	0 (0.0)	0.782
Cough = yes (%)	42 (77.8)	20 (71.4)	0.716
Fever = yes (%)	46 (85.2)	22 (78.6)	0.656
Comorbidity = yes (%)	8 (14.8)	16 (57.1)	< 0.001
Age	40.00 (28.25–50.00)	55.50 (44.75–65.75)	< 0.001
Blood routine test			
WBC (× 10^9^/L)	4.64 (4.04–5.64)	6.52 (5.13–7.25)	0.004
NEU%	62.50 (51.38–71.40)	80.50 (73.92–87.62)	< 0.001
NEU (× 10^9^/L)	2.69 (2.08–3.76)	5.21 (3.93–6.37)	< 0.001
MONO%	7.45 (5.55–8.57)	5.60 (4.45–7.95)	0.043
MONO (× 10^9^/L)	0.35 (0.26–0.45)	0.34 (0.24–0.56)	0.754
LYM%	27.45 (21.10–37.72)	12.90 (7.45–18.62)	< 0.001
LYM (× 10^9^/L)	1.29 (0.85–1.75)	0.82 (0.49–1.09)	< 0.001
RBC (× 10^12^/L)	4.62 (4.13–5.00)	4.51 (4.37–4.78)	0.685
Hemaglobin (g/L)	140.00 (122.00–153.75)	140.50 (129.00–144.50)	0.591
Hematocrit (%)	0.41 (0.37–0.46)	0.42 (0.39–0.43)	0.46
PLT (× 10^9^/L)	163.50 (135.50–224.25)	162.00 (128.75–202.75)	0.345
Coagulation test			
APTT (s)	38.70 (35.23–43.63)	35.80 (29.67–39.82)	0.019
TT (s)	13.90 (13.20–14.70)	14.45 (13.85–14.95)	0.045
Fibrinogen(g/L)	2.73 (1.88–3.40)	3.58 (3.19–4.31)	< 0.001
PT (s)	14.45 (13.43–16.38)	14.05 (13.28–14.95)	0.177
D-dimer (µg/L)	0.19 (0.08–0.28)	0.45 (0.23–0.66)	< 0.001
Clinical chemistry test			
TBIL (µmol/L)	14.20 (11.20–18.20)	14.75 (9.78–20.23)	0.728
DBIL (µmol/L)	5.30 (4.40–6.40)	5.80 (4.68–8.97)	0.096
ALT (U/L)	19.00 (14.00–35.00)	29.00 (23.75–42.25)	0.009
AST (U/L)	24.00 (20.00–34.00)	31.50 (23.00–39.50)	0.023
GGT (U/L)	22.00 (17.00–37.00)	42.00 (27.75–64.50)	< 0.001
ALP (U/L)	60.00 (48.00–71.00)	60.00 (42.75–81.25)	0.713
Total protein (g/L)	72.90 (68.60–75.80)	70.25 (66.75–73.45)	0.13
Albumin (g/L)	44.90 (42.10–48.20)	38.15 (36.00–40.85)	< 0.001
Glucose (mmol/L)	5.87 (5.34–6.82)	7.19 (6.43–9.83)	< 0.001
Urea (mmol/L)	3.59 (3.02–4.56)	5.71 (4.42–6.58)	< 0.001
Creatinine (µmol/L)	69.00 (59.00–82.00)	70.00 (59.50–76.25)	0.941
Uric acid (µmol/L)	259.00 (206.00–301.00)	204.50 (139.75–296.50)	0.068
Carbon dioxide (mmol/L)	25.30 (23.80–27.40)	25.70 (23.37–27.93)	0.728
Potassium (mmol/L)	3.99 (3.71–4.33)	3.97 (3.76–4.09)	0.538
Sodium (mmol/L)	138.00 (137.00–139.00)	137.00 (134.00–138.00)	0.002
Chlorine (mmol/L)	102.40 (100.90–104.00)	101.05 (97.95–102.82)	0.009
Calcium (mmol/L)	2.27 (2.18–2.32)	2.12 (2.08–2.19)	< 0.001
Phosphorus (mmol/L)	1.10 (0.97–1.20)	0.96 (0.89–1.14)	0.029
Magnesium (mmol/L)	0.87 (0.82–0.95)	0.92 (0.83–1.00)	0.06
Creatine kinase (U/L)	91.70 (55.10–150.10)	87.00 (61.05–167.65)	0.996
CKMB (U/L)	11.20 (9.10–15.80)	10.00 (9.00–13.00)	0.273
LDH (U/L)	205.00 (173.00–269.00)	284.00 (251.50–331.50)	< 0.001
Myohemoglobin (ng/mL)	30.00 (27.00–33.00)	36.00 (30.00–68.00)	0.004
Infection-related biomarkers			
Procalcitonin (ng/mL)	0.14 (0.10–0.16)	0.18 (0.13–0.23)	0.002
Interleukin-6 (pg/ml)	6.20 (5.25–6.83)	7.26 (6.66–9.91)	< 0.001
SAA (mg/L)	75.00 (18.43–175.40)	167.50 (118.50–209.88)	< 0.001
CRP (mg/L)	5.25 (2.00–21.93)	43.40 (14.52–96.05)	< 0.001

*WBC* white blood cell count, *LYM* lymphocyte count, *LYM%* lymphocyte percentage, *MONO* monocyte count, *MONO%* monocyte percentage, *NEU* neutrophils count, *NEU%* neutrophils percentage, *RBC* red blood cell count, *PLT* platelet count, *PT* prothrombin time, *APTT* activated partial thromboplastin time, *TT* thrombin time, *TBIL* total bilirubin, *DBIL* direct bilirubin, *AST* aspartate aminotransferase, *ALT* alanine aminotransferase, *GGT* gamma-glutamyltransferase, *ALP* alkaline phosphatase, *CKMB* creatine kinase isozyme, *LDH* lactic dehydrogenase, *SAA* serum amyloid a, *CRP* C-reactive protein

### Results of Sparse principal component analysis (SPCA)

When predicting the disease severity by multivariate logistic regression using clinical laboratory indicators directly, the fit curve did not converge. As the clinical laboratory markers always correlated with each other, we attempt to use SPCA to reduce dimensionality of the data and extract several PCs to explain such dozens of markers.

**Fig. 1:**
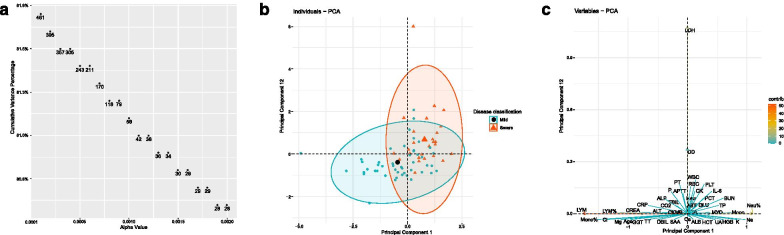
Results of the sparse principal components analysis using clinical data of Hefei cohort. Sparse principal analysis (SPCA) was performed based on the 44 clinical variables of Hefei cohort and the alpha parameter was adjusted from 0.0001 to 0.002 with stepsize 0.0001. For models of each alpha, the cumulative variance of the first 13 principal components (PCs) were summed and the number of variables selected in the first 13 PCs was counted. Variance of different alpha values in SPCA was plotted (**a**) and the number of selected clinical variables in the 13 PCs of each SPCA were added upon the point. **b** Distribution of the coronavirus disease 2019 (COVID-19) patients projected to principal components of SPCA with alpha of 0.0015. Depending on each patient's first (X-axis) and 12th (Y-axis) principal components value, COVID-19 patients were projected on the principal components plot of SPCA. **c** Scatter plot of the clinical markers selected in the first and 12th principal components of SPCA with alpha being 0.0015. Depending on each variable's first (X-axis) and 12th (Y-axis) principal components loadings, 44 clinical variables were projected on the principal components plot of SPCA. The first (X-axis) and 12^th^ (Y-axis) principal components accounted for the 17.8% and 2.9% of the total variance of the 44 clinical markers, respectively

Using sparsepca package, the SPCA was performed based on the 44 clinical variables and the alpha parameter was adjusted from 0.0001 to 0.002 with stepsize 0.0001. In such SPCA models, cumulative variance of the first 13 PCs were greater than 80% of the total variance. For models of each alpha, the cumulative variance of the first 13 PCs were summed and the number of variables selected in the first 13 PCs was counted (Fig. 1a). As alpha increases, the cumulative variance decreases gradually and the number of variables reduces sharply. When alpha is 0.0015, the first 13 PCs account for 80.8% of the cumulative variance of the original data and the number of variables selected in the 13 PCs is only 30. Based on the variance-sparsity trade-off [11], SPCA model with alpha of 0.0015 was used for further analysis.

The patients distribution and variables’ loadings using SPCA with alpha being 0.0015 were showed in Fig. 1b,c. The mild and severe ill COVID-19 patients distributed separately in the PC1 direction (X-axis) in the patients’ distribution plot (Fig. 1b). Each PC only depends on less than 5 clinical variables. An additional table file (Additional file 1: Table S1) shows this in more details.

Next, the 13 PCs were subjected to multivariate logistic regression for disease progression prediction. Using both step logistic regression and logistic regression with *L*_1_ penalty (glmnet package, https://cran.r-project.org/package=glmnet), two of the 13 PCs were finally selected in the prediction model, where the first PC (PC1) and the 12th PC (PC12) showed significant association with the disease severity classification (Table 2). This model was named as Model-A for further analysis.

**Table 2:** Multivariate logistic regression of 13 principal components produced by SPCA for disease severity prediction of COVID-19 patients

	Odd ratio	95% confidence interval	*P* value
PC1	4.049	1.126–21.015	0.057
PC2	0.563	0.239–1.940	0.182
PC3	1.446	0.791–2.938	0.256
PC4	0.749	0.300–1.501	0.478
PC5	3.486	1.057–14.825	0.059
PC6	1.730	0.572–5.837	0.344
PC7	0.675	0.285–1.347	0.296
PC8	1.635	0.652–12.627	0.486
PC9	0.793	0.298–2.051	0.624
PC10	3.329	1.122–13.325	0.053
PC11	0.712	0.317–1.548	0.390
PC12	3.318	1.260–11.949	0.041
PC13	0.898	0.405–1.970	0.783

The first to 13th principal components produced by SPCA were subjected to multivariate logistic regression for predict disease severity of COVID-19 patients. The first and 12th PCs finally showed significantly association with disease severity of COVID-19 patients

*COVID-19* coronavirus disease 2019, *PC* principal component, *SPCA* sparse principal component analysis

According to the PC loading matrix (Additional file 1: Table S1) and variable loading plots of SPCA (Fig. 1c), The PC1 depends on NEU%, LYM%, LYM, and MONO, while PC12 only depends on DD and LDH. Since the NEU%, LYM%, LYM, and MONO in PC1 could be obtained in one blood routine test and the PC1 accounted for 17.8% of the total variance, Model-A was further simplified to PC1, which was named as Lymphocyt-Monocyte-Neutrophil index, abbreviated as LMN index.

**Fig. 2:**
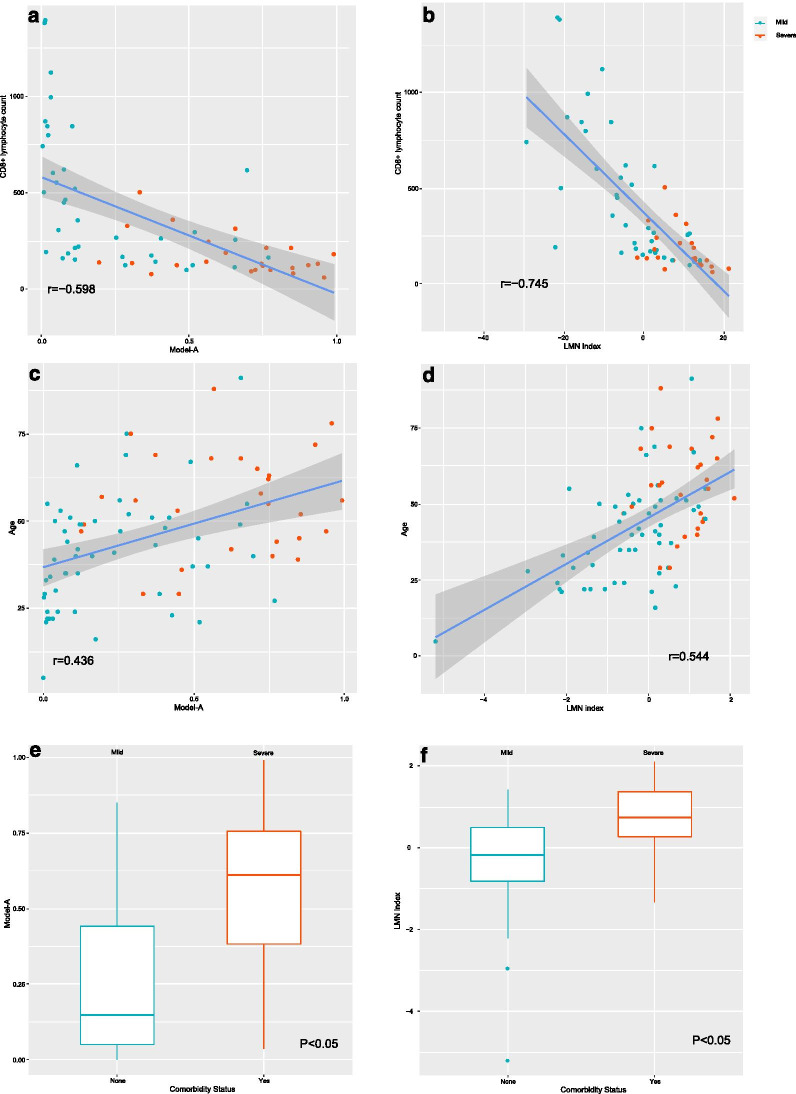
The association between prediction models with clinical characteristics of COVID-19 patients. Using prediction model Model-A and LMN index, in COVID-19 patients, CD8 + T lymphocytes negatively correlated with Model-A probability (**a**) and LMN index (**b**), while, patients age always positively correlated with Model-A probability (**c**) and LMN index (**d**). COVID-19 patients with comorbidity always have higher Model-A probabilities (**e**) and LMN index (**f**). Abbreviations: *COVID-19* coronavirus disease 2019, *Model-A* prediction model based on the first and 12th principal components produced by sparse principal component analysis, *LMN index* lymphocyte–monocyte–neutrophil index, a simplified version of Model-A

**Fig. 3:**
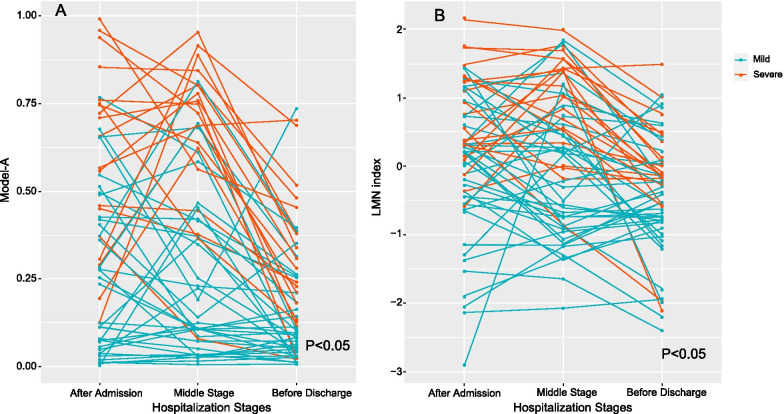
The dynamics of prediction models of COVID-19 patients from hospital admission to discharge. For all of the patients, results of three time points during hospitalization were collected: the first time point upon hospitalization (After Admission), the medium-term after hospitalization (Middle Stage), and the last time of laboratory test before hospital discharge (Before Discharge). With results of the three time points, the Model-A probability and LMN index were calculated and plotted. **a** Dynamics of Model-A probability for individual patients (P < 0.05); **b** dynamics of LMN index of individual patients (P < 0.05). Abbreviations: COVID-19, coronavirus disease 2019; Model-A, prediction model based on the first and 12th principal components produced by sparse principal component analysis; *LMN index* lymphocyte–monocyte–neutrophil index, a simplified version of Model-A

The relationships between Model-A and LMN index with clinical variables were assessed. Both of them showed significant correlation with CD8+ lymphocyte counts (Fig. 2a, b). Meanwhile, higher Model-A probabilities and LMN indices were observed in patients with comorbidities and older age (Fig. 2c–f). Furthermore, Model-A probabilities and LMN indices of different time point during hospitalization were investigated and both of them significantly decreased as treatment took effect and before discharge (*P* < 0.001, Fig. 3a, b). Patients with mild and severe status showed clearly variation tendency difference (*P* < 0.001) in Model-A probability and LMN index. Both of Model-A probabilities and the LMN indices of mild ill patients fell sharply (Fig. 3a, b, green lines), while the counterparts of severe ill patients declined slowly (Fig. 3a, b, red lines).

### Prediction efficiency evaluation

Then, ROC was used to estimate the disease severity classification performance of Model-A and the LMN index. The AUC and accuracy of Model-A for prediction of disease severity of COVID-19 patients were 0.867 and 0.726 in Hefei cohort (Table 3). The counterparts of LMN index were 0.837 and 0.793, respectively (Table 3).

**Table 3:** Summary of severity prediction efficiency of COVID-19 patients using Model-A, LMN index and other markers in Hefei cohort

	Cutoff	AUC(95% CI)	ACC(95% CI)	SEN(95% CI)	SPE(95% CI)	PPV(95% CI)	NPV(95% CI)
Model-A	0.125	0.867 (0.786–0.947)	0.726 (0.721–0.731)	1 (1–1)	0.574 (0.433–0.716)	0.565 (0.422–0.708)	1 (1–1)
LMN	0.281	0.837 (0.75–0.925)	0.793 (0.789–0.797)	0.786 (0.634–0.938)	0.796 (0.689–0.904)	0.667 (0.506–0.828)	0.878 (0.786–0.969)
ALB	42.05	0.846 (0.758–0.934)	0.79 (0.786–0.794)	0.821 (0.68–0.963)	0.774 (0.661–0.886)	0.657 (0.5–0.814)	0.891 (0.801–0.981)
CD4	352.5	0.773 (0.656–0.89)	0.721 (0.715–0.728)	0.75 (0.577–0.923)	0.703 (0.555–0.85)	0.621 (0.444–0.797)	0.812 (0.677–0.948)
CD8	146.5	0.809 (0.701–0.917)	0.77 (0.765–0.776)	0.625 (0.431–0.819)	0.865 (0.755–0.975)	0.75 (0.56–0.94)	0.78 (0.654–0.907)
CRP	7.6	0.811 (0.716–0.906)	0.683 (0.678–0.688)	0.964 (0.896–1.033)	0.537 (0.404–0.67)	0.519 (0.383–0.655)	0.967 (0.902–1.031)
DD	0.44	0.754 (0.641–0.868)	0.756 (0.752–0.761)	0.536 (0.351–0.72)	0.88 (0.79–0.97)	0.714 (0.521–0.908)	0.772 (0.663–0.881)
IL6	7.005	0.8 (0.691–0.909)	0.805 (0.801–0.809)	0.607 (0.426–0.788)	0.907 (0.83–0.985)	0.773 (0.598–0.948)	0.817 (0.719–0.915)
Lym	1.235	0.75 (0.641–0.86)	0.646 (0.641–0.652)	0.857 (0.728–0.987)	0.537 (0.404–0.67)	0.49 (0.35–0.63)	0.879 (0.767–0.99)
Lym%	21	0.833 (0.742–0.924)	0.793 (0.789–0.797)	0.857 (0.728–0.987)	0.759 (0.645–0.873)	0.649 (0.495–0.802)	0.911 (0.828–0.994)
Neu	3.825	0.786 (0.675–0.898)	0.768 (0.764–0.773)	0.786 (0.634–0.938)	0.759 (0.645–0.873)	0.629 (0.468–0.789)	0.872 (0.777–0.968)
Neu%	69.75	0.833 (0.745–0.922)	0.756 (0.752–0.761)	0.857 (0.728–0.987)	0.704 (0.582–0.825)	0.6 (0.448–0.752)	0.905 (0.816–0.994)
NLR	3.531	0.851 (0.765–0.937)	0.817 (0.814–0.821)	0.857 (0.728–0.987)	0.796 (0.689–0.904)	0.686 (0.532–0.84)	0.915 (0.835–0.995)

Disease severity prediction models: Model-A and LMN index were produced by SPCA and logistic regression from Hefei Cohort. Prediction efficiency of these models and clinical markers were assessed using ROC and AUC. ACC, SEN, SPE, PPV, NPV were calculated

*ALB* albumin, *AUC* area under curve, *CD4* CD4 + T lymphocytes, *CD8* CD8 + T lymphocytes, *CI* confidence interval, *COVID-19* coronavirus disease 2019, *CRP* C-reactive protein, *DD* D-Dimer, *IL6* interleukin-6, *LMN index* lymphocyte–monocyte–neutrophil index, simplified version of Model-A, *LYM* lymphocyte count, *LYM*% lymphocyte percentage, *Model-A* prediction model based on the first and 12th principal components produced by sparse principal component analysis, *NEU* neutrophil count, *NEU*%, eutrophils percentage, *NLR* netrophil-to-lymphocyte ratio, *NPV* negative predictive value, *PPV* positive predictive value, *NPV* negative predictive value, *PPV* positive predictive value

**Fig. 4:**
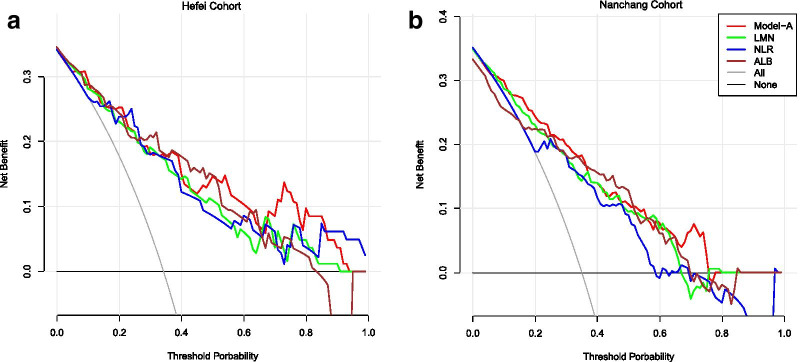
Decision curve analysis of prediction models produced by SPCA. Decision curve analysis of prediction models in the training Hefei Cohort (**a**) and independent validation Nanchang Cohort (**b**). Model-A showed slightly better net benefit both in Hefei Cohort and Nanchang Cohort. Abbreviations: *ALB* Albumin, *COVID-19* coronavirus disease 2019, *Model-A* prediction model based on the first and 12th principal components produced by sparse principal component analysis, *LMN index* lymphocyte–monocyte–neutrophil index, a simplified version of Model-A, *NLR* Neutrophil-to-lymphocyto ratio

Since several laboratory markers are classical predictors of disease severity, so we also compared the prediction results of these markers and they were summarized in Table 3. The Model-A showed the best performance and LMN index showed robust prediction effect compared with classical predictors including neutrophil-to-lymphocyte ratio (NLR) which is a hopeful predictor for severity ill COVID-19 [12, 13]. In order to assess the clinical net benefit of Model-A and LMN index, we also performed decision curve analysis (Fig. 4a). Although curves of all the markers tangled and the Model-A gave slightly greater net benefit, while the LMN index just showed similar performance as albumin and NLR.

### Independent cohort validation

In order to validate the severity prediction efficiency of Model-A and LMN index, the laboratory indicators of Nanchang Cohort (An additional table file shows this in more detail in Additional file 1: Table S2) were scaled and PC1 and PC12 of each patient were calculated using PC loading matrix of Hefei Cohort. Model-A probabilities and LMN indices were then predicted. ROC estimated the severity prediction efficiency, and the AUC and accuracy of Model-A produced with Nanchang Cohort for disease progression prediction were 0.835 and 0.757, respectively (Table 4). Meanwhile, the AUC and accuracy of LMN index were 0.800 and 0.740 in this independent cohort. Just like in the training cohort (Table 3). Model-A also gave the best efficiency and the LMN index showed comparable prediction efficiency compared with classical clinical variables (Table 4), such as NLR, albumin and so on. In decision curve analysis (Fig. 4b), all the curves intertwined and the Model-A gave slightly better performance, while the LMN index just showed similar performance as albumin and NLR.

**Table 4:** Summary of severity prediction efficiency of COVID-19 patients in the independent cohort (Nanchang Cohort)

	Cutoff	AUC(95% CI)	AUC(95% CI)	SEN(95% CI)	SPE(95% CI)	PPV(95% CI)	NPV(95% CI)
Model-A	0.484	0.835 (0.774–0.895)	0.835 (0.774–0.895)	0.831 (0.735–0.926)	0.718 (0.634–0.802)	0.612 (0.506–0.719)	0.888 (0.822–0.953)
LMN	0.711	0.8 (0.733–0.868)	0.8 (0.733–0.868)	0.729 (0.615–0.842)	0.745 (0.664–0.827)	0.606 (0.492–0.719)	0.837 (0.764–0.91)
ALB	38.25	0.816 (0.742–0.891)	0.816 (0.742–0.891)	0.769 (0.655–0.884)	0.788 (0.71–0.867)	0.645 (0.526–0.764)	0.872 (0.805–0.94)
CD4	308	0.708 (0.624–0.791)	0.708 (0.624–0.791)	0.625 (0.498–0.752)	0.731 (0.646–0.816)	0.556 (0.433–0.678)	0.784 (0.702–0.865)
CD8	176	0.694 (0.608–0.781)	0.694 (0.608–0.781)	0.554 (0.423–0.684)	0.75 (0.667–0.833)	0.544 (0.415–0.673)	0.757 (0.674–0.84)
CRP	8.15	0.7 (0.618–0.781)	0.7 (0.618–0.781)	0.724 (0.609–0.839)	0.578 (0.485–0.671)	0.477 (0.373–0.582)	0.797 (0.709–0.886)
DD	0.99	0.758 (0.675–0.841)	0.758 (0.675–0.841)	0.615 (0.483–0.748)	0.823 (0.747–0.899)	0.653 (0.52–0.786)	0.798 (0.719–0.877)
Lym	0.605	0.785 (0.714–0.855)	0.785 (0.714–0.855)	0.593 (0.468–0.719)	0.872 (0.809–0.934)	0.714 (0.588–0.841)	0.798 (0.726–0.87)
Lym%	13.95	0.788 (0.716–0.859)	0.788 (0.716–0.859)	0.746 (0.635–0.857)	0.743 (0.661–0.825)	0.611 (0.499–0.724)	0.844 (0.771–0.916)
Neu	5.055	0.71 (0.625–0.794)	0.71 (0.625–0.794)	0.729 (0.615–0.842)	0.661 (0.572–0.749)	0.538 (0.428–0.647)	0.818 (0.738–0.899)
Neu%	78.4	0.775 (0.701–0.849)	0.775 (0.701–0.849)	0.763 (0.654–0.871)	0.706 (0.621–0.792)	0.584 (0.474–0.694)	0.846 (0.772–0.92)
NLR	5.691	0.784 (0.711–0.856)	0.784 (0.711–0.856)	0.746 (0.635–0.857)	0.743 (0.661–0.825)	0.611 (0.499–0.724)	0.844 (0.771–0.916)

Disease severity prediction models: Model-A and LMN index were produced by SPCA and logistic regression from Hefei Cohort. Prediction efficiency of these models and clinical markers were assessed in independent validation cohort using ROC and AUC. ACC, SEN, SPE, PPV, NPV were calculated

*ALB* albumin, *AUC* area under curve, *CD4* CD4 + T lymphocytes, *CD8* CD8 + T lymphocytes, *CI* confidence interval, *COVID-19* coronavirus disease 2019, *CRP* C-reactive protein, *DD* D-Dimer, *LMN index* lymphocyte–monocyte–neutrophil index, simplified version of Model-A, *LYM* lymphocyte count, *LYM%* lymphocyte percentage, *Model-A* prediction model based on the first and 12th principal components produced by sparse principal component analysis, *NEU* neutrophil count, *NEU%* neutrophils percentage, *NLR* netrophil-to-lymphocyte ratio, *NPV* negative predictive value, *PPV* positive predictive value, *NPV* negative predictive value, *PPV* positive predictive value

## Discussion

Since the outbreak of COVID-19, the number of patients worldwide has increased drastically, which put massive pressure on the health care system of every country. In order to save lives as more as possible, more resources should be focused on the severe ill patients. Several studies have attempted to seek the predictors of disease progression of COVID-19, such as Neutrophil-to-lymphocyte ratio [12, 13], thrombocytopenia [5], DD, IL-6 [7] and so on. There are also dozens of laboratory indicators used for disease severity prediction. In present study, we used SPCA to extract principal components of laboratory indicators. In SPCA model with alpha being 0.0015, the first 13 PCs accounted 80.8% of the total variance of the 44 clinical variables. Using logistic regression, Model-A based on PC1 and PC12 was deduced and showed the best prediction efficiency in the training cohort (Hefei Cohort. AUC = 0.867) as well as the independent validation cohort (Nanchang Cohort. AUC = 0.835). Because PC1 depending on blood routine test markers accounted 17.8% of the total variance, Model-A was further simplified to LMN index, which predicted disease severity just using PC1. LMN index also showed satisfactory prediction efficiency in the Hefei Cohort (AUC = 0.837) as well as the independent Nanchang Cohort (AUC = 0.800). In decision curve analysis, Model-A showed slightly better performance both in the Hefei Cohort and Nanchang Cohort and the LMN index performed comparably to albumin and NLR.

In clinical laboratory, combinations of test items are very common, while indicators in these combinations always correlated with each other. Such as in blood routine examination, the neutrophil counts always negatively relate with lymphocyte counts and in liver function examination, serum ALT always changes in parallel with AST alteration. This feature of laboratory markers is called collinearity and could enhance the diagnostic accuracy. The collinearity of these laboratory markers makes it difficult for traditional multivariate statistical analysis to include all the significant indicators. This is why PCA is used in this study, which can extract distinct PC from a group of highly correlated variables in combinations of the original variables [14, 15]. Furthermore, controlling parameter alpha was induced to PCA for better variable selection, which is the so-called SPCA [16, 17]. In this study, alpha value was adjusted from 0.0001 to 0.002 and when alpha was set as 0.0015, the 13 PCs accounted for 80.8% of the total variance of the 44 clinical variables and only depended on 30 variables. Thus this SPCA model balanced variance and sparsity [11] and could represent the original 44 variables. Furthermore, sparsepca package [9] used in current study is a recently published method for SPCA, which offers some immediate improvements over previously proposed SPCA algorithms, such as much faster and more scalable algorithm, robustness to outliers.

In the disease severity prediction Model-A, the PC1 is dependent on four clinical markers: NEU%, MONO, LYM%, and LYM, while the PC12 merely depends on DD and LDH. Several previous studies have convinced the relationship between LYM decrease and NEU increase in severe ill COVID-19, SARS and MERS patients [18–23]. While in this research, both the cell counts and percentage of lymphocyte showed importance in disease progression. Several studies have also confirmed that severe ill COVID-19 patients always accompany with higher DD [7, 19] and LDH [7, 19, 23]. So Model-A may represent inflammation status, coagulation status, metabolism status of COVID-19 patients. Alteration in inflammation response, coagulation system and metabolism even hypoxia in COVID-19 patients could result in Model-A probability change. So that’s why Model-A combined all these markers give the best performance in ROC and decision curve analysis for disease severity prediction.

Furthermore, monocyte may also play roles in the disease progression which was less noticed before and need further investigations. On the other hand, because the range of numeric values of different variables varied widely and variables with larger numeric values would dominate analysis, monocyte, which shows significance in this research, is rarely concerned in previous studies. So the process of data standardization is critical in multivariate analysis.

Furthermore, we also found Model-A and LMN index were significantly associated with age, comorbidity status and CD8+ T cells. It is particularly important that Model-A probability and LMN index change significantly during COVID-19 patients hospitalization and they decrease obviously as treatment takes effect. Meanwhile, in moderate ill patients, they decreased more sharply than counterparts in severe ill patients. In 7 of 28 severe ill patients, Model-A first rose then descended and only in 7 of 54 mild ill ones, Model-A showed the same tendency. These evidence showed that continuous surveillance of Model-A and LMN index during treatment may have special clinical importance.

The prediction efficiency of Model-A and LMN index for disease severity is encouraging. The Model-A showed the best prediction efficiency both in training cohort and independent validation cohort. While, the LMN index also gives the AUC of 0.837 and 0.800 in training data and validation data respectively, which performed better than classical markers including LYM%, NEU%, CRP, IL6, etc. Even compared with the NLR, which is recently reported as a hopeful predictor of inflammation or severity, LMN index still showed the better prediction value in the independent cohort (AUC: 0.800 VS 0.784). Though AUC of LMN index in training cohort is smaller than Alb and NLR, in clinical setting, LMN index depending on more variables may perform more robust than Alb or NLR, both of which are more susceptible to physical and pathological changes.

The NPV of Model-A was 1 in training cohort and 0.888 in validation cohort, respectively, so COVID-19 patients with the Model-A probability smaller than cutoff point may have little probability of developing to severe cases. The LMN also showed great NPV both in training cohort and validation cohort. So with these evaluation approaches, health care staff could monitor present COVID-19 patients and focus on the cases with more risk of progression earlier, which will benefit to save more lives.

For evaluation of net benefit of the models, decision curve analysis was also performed, and Model-A still show slightly better performance than Alb and NLR. According to previous study [24], a little improvement is also improvement, so Model-A indeed bring net benefit for patients. While in DCA, LMN index just showed comparable performance as Alb and NLR.

Finally, Model-A shows the best prediction efficiency for disease severity of COVID-19 patients, and the LMN index depending on four blood routine test markers. is very economical for clinical application. So both of them have the potential for clinical use in COVID-19 treatment and even in other disease treatment. This use of SPCA for clinical variables extraction may also shadow new application direction of SPCA.

Our study also have some weaknesses. Clinical characteristics other than laboratory markers were not concerned in this study, which were also risk factors of disease progression. More clinical characteristics should be included for model training in future. On the other hand, the sample size was small, which may have some impact on the statistical results and bias may exist during data standardization process, model training and cut-point selection. In future, with numerous patients enrolled to optimize the above processes, more accurate prediction model will be produced.

## Conclusions

In the study, using SPCA method for feature selection and dimensionality reduction, prediction model Model-A and LMN index were deduced, which showed significant association with clinical outcomes and robust disease severity prediction efficiency of COVID-19 patients. Model-A and LMN index may have the potential for clinical application and are helpful to the patients classification so as to save more lives.

## Supplementary Information

**Additional file 1. Lymphocyte–monocyte–neutrophil index: a predictor of severity of coronavirus disease 2019 patients produced by sparse principal component analysis.** **Table S1:** Principal component loadings for thirteen principal components produced by sparse principal component analysis. **Table S2**: Demographics and baseline laboratory markers of patients in the independent cohort (NanChang Cohort).

## Data Availability

Not applicable.

## Abbreviations

COVID-19: Coronavirus disease 2019
SARS-CoV-2: Severe acute respiratory syndrome coronavirus 2
SPCA: Sparse principal component analysis
PCs: Principal components
ROC: Receiver operator characteristic curve
PPV: Positive predictive value
NPV: Negative predictive value
WBC: White blood cell count
NEU: Neutrophil count
NEU%: Neutrophil percentage
AST: Aspartate aminotransferase
ALT: Alanine aminotransferase
GGT: Gamma-glutamyltransferase
Glu: Glucose
LDH: Lactic dehydrogenase
SAA: Serum amyloid a
CRP: C-reactive protein
PCT: Procalcitonin
IL-6: Interleukin-6
DD: D-Dimer
MYO: Myohemoglobin
LYM: Lymphocyte count
LYM%: Lymphocyte percentage
Alb: Albumin
Ca: Calcium
P: Phosphorus
FIB: Fibrinogen
NLR: Neutrophil-to-lymphocyte ratio
AUC: Area under curve
DCA: Decision curve analysis
LMN index: Lymphocyte–monocyte–neutrophil index, simplified version of Model-A
Model-A: Prediction model based on the first and 12th principal components produced by sparse principal component analysis
