# Supplementary material for: Lymphocyte–monocyte–neutrophil index: a predictor of severity of coronavirus disease 2019 patients produced by sparse principal component analysis
Source: Virol J. 2021 Jun 4;18:115. doi: 10.1186/s12985-021-01561-9 (PMC8176446; doi:10.1186/s12985-021-01561-9)
Supplement: Supplementary file 1 — Additional file 1. Lymphocyte–monocyte–neutrophil index: a predictor of severity of coronavirus disease 2019 patients produced by sparse principal component analysis. Table S1: Principal component loadings for thirteen principal components produced by sparse principal component analysis. Table S2: Demographics and baseline laboratory markers of patients in the independent cohort (NanChang Cohort). [file 12985_2021_1561_MOESM1_ESM.doc]

Table S1. Principal component loadings for thirteen principal components produced by of sparse principal component analysis of 44 clinical indicators (Alpha=0.0015)

|  | PC1 | PC2 | PC3 | PC4 | PC5 | PC6 | PC7 | PC8 | PC9 | PC10 | PC11 | PC12 | PC13 |
| --- | --- | --- | --- | --- | --- | --- | --- | --- | --- | --- | --- | --- | --- |
| Age | 0 | 0 | 0 | 0 | 0 | 0 | 0 | 0 | 0 | 0 | 0 | 0 | 0 |
| WBC | 0 | 0 | 0 | -0.815 | 0 | 0 | 0 | 0 | 0 | 0 | 0 | 0 | 0 |
| NEU% | 0.386 | 0 | 0 | -0.011 | 0 | 0 | 0 | 0 | 0 | 0 | 0 | 0 | 0 |
| NEU | 0 | 0 | 0 | -0.563 | 0 | 0 | 0 | 0 | 0 | 0 | 0 | 0 | 0 |
| MONO% | 0 | 0 | 0 | 0 | 0 | 0 | 0 | 0 | 0 | 0 | 0 | 0 | -0.388 |
| MONO | 0.133 | 0 | 0 | -0.165 | 0 | 0 | 0 | 0 | 0 | 0 | 0 | 0 | 0 |
| LYM% | -0.277 | 0 | 0 | 0.009 | 0 | 0 | 0 | 0 | 0 | 0 | 0 | 0 | 0 |
| LYM | -0.620 | 0 | 0 | 0 | 0 | 0 | 0 | 0 | 0 | 0 | 0 | 0 | 0 |
| RBC | 0 | 0 | 0.029 | 0 | 0 | 0 | 0 | 0 | 0 | 0 | 0 | 0 | 0 |
| Hemaglobin | 0 | 0 | 0.734 | 0 | 0 | 0 | 0 | 0 | 0 | 0 | 0 | 0 | 0 |
| Hematocrit | 0 | 0 | 0.660 | 0 | 0 | 0 | 0 | 0 | 0 | 0 | 0 | 0 | 0 |
| PLT | 0 | 0 | 0 | 0 | 0 | 0 | 0 | 0 | 0 | 0 | 0 | 0 | 0 |
| APTT | 0 | 0 | 0 | 0 | 0 | 0 | 0 | 0 | 0 | 0 | 0 | 0 | 0 |
| TT | 0 | 0 | 0 | 0 | 0 | 0 | 0 | 0 | 0 | 0 | 0 | 0 | 0.909 |
| Fibrinogen | 0 | 0 | 0 | 0 | 0 | 0.026 | 0 | 0 | 0 | 0 | 0 | 0 | 0 |
| PT | 0 | 0 | 0 | 0 | 0 | 0 | 0 | 0 | 0 | -0.989 | 0 | 0 | 0 |
| D-dimer | 0 | 0 | 0 | 0 | 0 | 0 | 0 | 0.927 | 0 | 0 | 0 | 0.327 | 0 |
| Procalcitonin | 0 | 0 | 0 | 0 | 0 | 0 | 0 | 0 | 0 | 0 | 0 | 0 | 0 |
| Interleukin-6 | 0 | 0 | 0 | 0 | 0 | 0 | 0 | 0 | 0 | 0 | -0.043 | 0 | 0 |
| SAA | 0 | 0 | 0 | 0 | 0 | 0 | 0 | 0 | 0 | 0 | 0 | 0 | 0 |
| CRP | 0 | 0 | 0 | 0 | 0 | 0 | 0 | 0 | 0 | 0 | 0 | 0 | 0 |
| TBIL | 0 | 0 | 0 | 0 | 0.841 | 0 | 0 | 0 | 0 | 0 | 0 | 0 | 0 |
| DBIL | 0 | 0 | 0 | 0 | 0.399 | 0 | 0 | 0 | 0 | 0 | 0 | 0 | 0 |
| ALT | 0 | 0 | 0 | 0 | 0 | 0 | 0 | 0 | 0 | 0 | 0 | 0 | 0 |
| AST | 0 | 0 | 0 | 0 | 0 | 0 | 0 | 0 | 0 | 0 | 0 | 0 | 0 |
| GGT | 0 | 0 | 0 | 0 | 0 | 0 | 0 | 0 | 0 | 0 | 0 | 0 | 0 |
| ALP | 0 | 0 | 0 | 0 | 0 | 0 | 0 | 0 | 0.996 | 0 | 0 | 0 | 0 |
| Total protein | 0 | 0 | 0 | 0 | 0 | 0 | 0 | 0 | 0 | 0 | 0.982 | 0 | 0 |
| Albumin | 0 | 0 | 0 | 0 | 0 | 0 | 0 | 0 | 0 | 0 | 0 | 0 | 0 |
| Glucose | 0 | 0 | 0 | 0 | 0 | 0 | 0 | 0 | 0 | 0 | 0 | 0 | 0 |
| Urea | 0 | 0 | 0 | 0 | 0 | 0 | 0 | 0 | 0 | 0 | 0 | 0 | 0 |
| Creatinine | 0 | -0.776 | 0 | 0 | 0 | 0 | 0 | 0 | 0 | 0 | 0 | 0 | 0 |
| Uric acid | 0 | 0 | 0 | 0 | 0 | 0 | 0 | 0 | 0 | 0 | 0 | 0 | -0.115 |
| Carbon dioxide | 0 | 0 | 0 | 0 | 0 | 0 | 0 | 0 | 0 | 0 | 0 | 0 | 0 |
| Potassium | 0 | 0 | 0 | 0 | 0 | 0 | 0 | 0 | 0 | 0 | 0 | 0 | 0 |
| Sodium | 0 | 0 | 0 | 0 | 0 | 0 | 0 | 0 | 0 | 0 | 0 | 0 | 0 |
| Chlorine | 0 | 0 | 0 | 0 | 0 | 0 | 0 | 0 | 0 | 0 | 0 | 0 | 0 |
| Calcium | 0 | 0 | 0 | 0 | 0 | 0 | 0 | 0 | 0 | 0 | 0 | 0 | 0 |
| Phosphorus | 0 | 0 | 0 | 0 | 0 | -0.981 | 0 | 0 | 0 | 0 | 0 | 0 | 0 |
| Magnesium | 0 | 0 | 0 | 0 | 0 | 0 | 0 | 0 | 0 | 0 | 0 | 0 | 0 |
| Creatine kinase | 0 | 0 | 0 | 0 | 0 | 0 | 1.008 | 0 | 0 | 0 | 0 | 0 | 0 |
| CKMB | 0 | 0 | 0 | 0 | 0 | 0 | 0 | 0 | 0 | 0 | 0 | 0 | 0 |
| LDH | 0 | 0 | 0 | 0 | 0 | 0 | 0 | -0.314 | 0 | 0 | 0 | 0.933 | 0 |
| Myohemoglobin | 0 | -0.579 | 0 | 0 | 0 | 0 | 0 | 0 | 0 | 0 | 0 | 0 | 0 |

The first to 13th principal components accounted for 80.8% of total variance of the 44 clinical markers and principal component loadings of them were presented.

Abbreviations: PC, principal component; WBC, white blood cell count; LYM, lymphocyte count; LYM%, lymphocyte percentage; MONO, monocyte count; MONO%, monocyte percentage; NEU, neutrophils count; NEU%, neutrophils percentage ; RBC, red blood cell count ; PLT, platelet count; PT, prothrombin time ; APTT, activated partial thromboplastin time ; TT, thrombin time; TBIL, total bilirubin ; DBIL, direct bilirubin; AST, aspartate aminotransferase ; ALT, alanine aminotransferase; GGT, gamma-glutamyltransferase; ALP, alkaline phosphatase ; CKMB, creatine kinase isozyme ; LDH, lactic dehydrogenase ; SAA, serum amyloid a; CRP, C-reactive protein;

Table S2. Demographics and baseline laboratory markers of mild and severe ill COVID-19 patients in the independent cohort (Nanchang Cohort)

|  | Mild  (n=110) | Severe  (n=59) | *P* |
| --- | --- | --- | --- |
| Hypertension= Yes (%) | 11 (10.0) | 19 ( 32.2) | 0.001 |
| diabetes = Yes (%) | 15 (13.6) | 17 ( 28.8) | 0.028 |
| length_of_stay (mean (SD)) | 13.41 (6.22) | 19.92 (7.38) | <0.001 |
| gender = male (%) | 60 (54.5) | 41 ( 69.5) | 0.085 |
| Age | 43.50 (33.00- 51.00) | 52.00 (43.00- 66.00) | <0.001 |
| Blood Routine Test |  |  |  |
| WBC (×10^9^/L) | 5.89 (4.22- 7.47) | 7.37 (5.88- 10.85) | 0.001 |
| NEU% | 70.80 (60.10- 80.00) | 88.30 (78.75- 92.35) | <0.001 |
| NEU (×10^9^/L) | 4.01 (2.75- 5.52) | 6.21 (4.71- 9.74) | <0.001 |
| MONO% | 6.50 (4.90- 8.10) | 4.50 (2.95- 7.10) | 0.001 |
| MONO (×10^9^/L) | 0.38 (0.26- 0.46) | 0.37 (0.24- 0.52) | 0.772 |
| LYM% | 20.30 (13.70- 30.50) | 7.30 (4.50- 14.70) | <0.001 |
| LYM (×10^9^/L) | 1.11 (0.78- 1.56) | 0.55 (0.41- 0.96) | <0.001 |
| RBC (×10^12^/L) | 4.60 (4.25- 4.90) | 4.32 (3.89- 4.70) | 0.006 |
| Hemaglobin (g/L) | 144.00 (134.00- 152.00) | 136.00 (122.50- 146.00) | 0.006 |
| Hematocrit (%) | 0.42 (0.38- 0.44) | 0.39 (0.35- 0.41) | 0.002 |
| PLT (×10^9^/L) | 203.00 (161.00- 247.00) | 177.00 (137.50- 258.00) | 0.152 |
| Coagulation Test |  |  |  |
| APTT (Second) | 28.00 (26.45- 30.52) | 28.40 (25.45- 31.60) | 0.777 |
| TT (Second) | 16.40 (15.80- 17.30) | 16.70 (15.88- 18.38) | 0.186 |
| Fibrinogen(g/L) | 3.51 (2.75- 4.59) | 3.92 (3.15- 5.02) | 0.068 |
| PT (Second) | 12.30 (11.78- 12.90) | 12.75 (11.90- 13.53) | 0.06 |
| D-dimer (µg/L) | 0.38 (0.21- 0.78) | 1.16 (0.55- 4.10) | <0.001 |
| Clinical Chemistry Test |  |  |  |
| TBIL (µmol/L) | 11.65 (7.85- 16.38) | 12.65 (8.30- 16.25) | 0.534 |
| DBIL (µmol/L) | 3.80 (2.38- 5.23) | 4.35 (2.98- 6.60) | 0.103 |
| ALT (U/L) | 17.00 (12.00- 28.00) | 29.00 (17.00- 46.25) | <0.001 |
| AST (U/L) | 19.50 (16.75- 24.25) | 28.50 (21.00- 37.00) | <0.001 |
| GGT (U/L) | 21.00 (13.00- 36.25) | 37.00 (26.00- 85.25) | <0.001 |
| ALP (U/L) | 62.00 (49.75- 72.00) | 71.50 (60.00- 83.50) | 0.002 |
| Total protein (g/L) | 68.85 (65.20- 73.35) | 66.15 (62.02- 73.40) | 0.097 |
| Albumin (g/L) | 41.90 (38.77- 46.02) | 34.55 (31.80- 38.12) | <0.001 |
| Glucose (mmol/L) | 6.80 (5.48- 8.47) | 10.71 (8.02- 12.40) | <0.001 |
| Urea (mmol/L) | 4.00 (3.40- 5.00) | 6.10 (4.30- 7.93) | <0.001 |
| Creatinine (µmol/L) | 62.60 (52.80- 78.53) | 65.45 (56.35- 82.35) | 0.226 |
| Uric acid (µmol/L) | 241.00 (196.50- 292.00) | 249.00 (158.00- 308.50) | 0.575 |
| Potassium (mmol/L) | 3.73 (3.49- 4.20) | 4.06 (3.48- 4.50) | 0.03 |
| Sodium (mmol/L) | 136.60 (134.45- 137.78) | 135.35 (131.93- 137.12) | 0.021 |
| Chlorine (mmol/L) | 99.75 (96.93- 101.45) | 98.20 (94.77- 100.95) | 0.088 |
| Calcium (mmol/L) | 1.98 (1.92- 2.09) | 1.87 (1.80- 1.95) | <0.001 |
| Creatine kinase (U/L) | 65.00 (46.25- 103.00) | 80.00 (42.25- 191.50) | 0.477 |
| CKMB (U/L) | 11.00 (9.00- 15.00) | 14.00 (11.00- 19.75) | 0.006 |
| Myohemoglobin (ng/mL) | 27.00 (21.00- 39.20) | 45.05 (30.48- 80.33) | <0.001 |
| Infection-related biomarkers |  |  |  |
| Procalcitonin (ng/mL) | 0.04 (0.02- 0.06) | 0.06 (0.04- 0.11) | <0.001 |
| CRP (mg/L) | 6.40 (1.38- 23.14) | 20.45 (7.18- 68.36) | <0.001 |

Abbreviations: COVID-19, coronvirus disease 2019; WBC, white blood cell count; LYM, lymphocyte count; LYM%, lymphocyte percentage; MONO, monocyte count; MONO%, monocyte percentage; NEU, neutrophils count; NEU%, neutrophils percentage ; RBC, red blood cell count ; PLT, platelet count; PT, prothrombin time ; APTT, activated partial thromboplastin time ; TBIL, total bilirubin ; DBIL, direct bilirubin; AST, aspartate aminotransferase ; ALT, alanine aminotransferase; GGT, gamma-glutamyltransferase; ALP, alkaline phosphatase ; CKMB, creatine kinase isozyme ; CRP, C-reactive protein;
